# In-Vivo Antidiabetic Activity and In-Silico Mode of Action of LC/MS-MS Identified Flavonoids in Oleaster Leaves

**DOI:** 10.3390/molecules25215073

**Published:** 2020-11-01

**Authors:** Hamza Mechchate, Imane Es-Safi, Mohammed Bourhia, Andrii Kyrylchuk, Abdelfattah El Moussaoui, Raffaele Conte, Riaz Ullah, Essam Ezzeldin, Gamal A. Mostafa, Andriy Grafov, Hicham Bekkari, Dalila Bousta

**Affiliations:** 1 Laboratory of Biotechnology, Environment, Agrifood, and Health, Sidi Mohamed Ben Abdellah University, Fez 30050, Morocco; hamza.mechchate@usmba.ac.ma (H.M.); imane.essafi1@usmba.ac.ma (I.E.-S.); abdelfattah.elmoussaoui@usmba.ac.ma (A.E.M.); Hicham.bekkari@usmba.ac.ma (H.B.); boustadlila@gmail.com (D.B.); 2 Laboratory of Chemistry-Biochemistry, Environment, Nutrition, and Health, Faculty of Medicine and Pharmacy, University of Casablanca, B.P 5696, Casablanca 20250, Morocco; 3 Institute of Organic Chemistry, National Academy of Sciences of Ukraine, Murmanska Street 5, 02660 Kyiv, Ukraine; iamkaant@gmail.com; 4 Research Institute on Terrestrial Ecosystems (IRET)—CNR, Via Pietro Castellino 111, 80131 Naples, Italy; Raffaele.conte86@tiscali.it; 5 Department of Pharmacognosy, College of Pharmacy, King Saud University, Riyadh 11451, Saudi Arabia; rullah@ksu.edu.sa; 6 Department of Pharmaceutical Chemistry, College of Pharmacy King Saud University, Riyadh 11451, Saudi Arabia; esali@ksu.edu.sa (E.E.); gmostafa@ksu.edu.sa (G.A.M.); 7 Department of chemistry, University of Helsinki, P.O. Box 55, FI-00014 Helsinki, Finland; andriy.grafov@helsinki.fi

**Keywords:** *Olea europea* L. subsp. *europaea* var. *sylvestris* (Mill) Lehr, oleaster, diabetes, flavonoids, molecular docking

## Abstract

Background: *Olea europea* L. subsp. *europaea* var. *sylvestris* (Mill) Lehr (Oleaster) is a wild endemic olive tree indigenous to the Mediterranean region. *Olea europea* leaves represent a natural reservoir of bioactive molecules that can be used for therapeutic purposes. Aim of the study: This work was conducted to study antidiabetic and antihyperglycemic activities of flavonoids from oleaster leaves using alloxan-induced diabetic mice. The mode of action of flavonoids against eight receptors that have a high impact on diabetes management and complication was also investigated using molecular docking. Results: During 28 days of mice treatment with doses 25 and 50 mg/kg b.w, the studied flavonoids managed a severe diabetic state (<450 mg/dL), exhibiting a spectacular antidiabetic and antihyperglycemic activity, and improved mice health status compared to diabetic control. The in-silico mode of action of oleaster flavonoids revealed the inhibition of protein tyrosine phosphatase 1B (PTP1B), Dipeptidyl-peptidase 4 (DPP4), α-Amylase (AAM), α-Glucosidase inhibition, Aldose reductase (AldR), Glycogen phosphorylase (GP), and the activation of free fatty acid receptor 1 (FFAR1). Conclusion: The findings obtained in the present work indicate that the flavonoids from the oleaster may constitute a safe multi-target remedy to treat diabetes.

## 1. Introduction

Diabetes is the most common metabolic disease worldwide with several underlying causes leading to hyperglycemia [1]. With two main types, type 1 diabetes is characterized by autoimmune-mediated destruction of pancreatic beta cells, which affect the normal production of insulin, the key hormone regulating blood glucose (5% of all diabetes types). In contrast, type 2 diabetes occurs by a resistance to insulin action or insulin deficiency (90% type 2 diabetes). The left 5% represents other subtypes [2].

The number of new cases is rising. The International Diabetes Federation expects over 700 Million cases by 2045 against 425 million recorded in 2017 [3]. The scientific community developed during the years many chemical and synthetic antidiabetic drugs to help manage the diabetic state and to prevent the complications [4]. Still, the adverse effect of their prolonged administration (such as gastrointestinal disorders, hypoglycemia, obesity) have changed the attention of many people and researchers for new safer alternatives [5]. Many diabetic people have changed their medication into medicinal plants as effective, safe, and present little or almost no side effect [6].

*Olea europaea* subsp. *europaea* L. including two varieties: cultivated and wild species, respectively called *europaea* (cultivars) and *sylvestris* (oleaster), which are naturally distributed all around the Mediterranean region [7]. The medicinal properties of oleaster described in the antique books are mostly attributed to the leaves, which are used in natural remedies with infusion or decoction [8]. In Morocco, they were described by the population as a traditional treatment for both type 1 and type 2 diabetes [9].

In this work, we investigated the antidiabetic activity and the mode of action through molecular docking of flavonoids from oleaster leaves (*Olea europea* L. subsp. *europaea* var. *sylvestris* (Mill) Lehr).

## 2. Materials and Methods

### 2.1. Chemicals and Reagents

Alloxan Monohydrate, D-glucose were purchased from Sigma-Aldrich (St. Louis, MO, USA), Glibenclamide was obtained from a local pharmacy. Reagent grade chemicals were used to perform this study.

### 2.2. Plant Material

*Olea europea* L. subsp. *europaea* var. *sylvestris* (Mill) Lehr, leaves were collected in April 2019 from the region of Taounate (Latitude: 34°32′9″, Longitude: 4°38′24″, Altitude: 566 m) located at the North of Morocco. The plant was taxonomically authenticated under #BPRN57, and the voucher specimen has been deposited at the herbarium of the Laboratory of Biotechnology, Environment, Agrifood, and Health in the Faculty of Sciences Dhar el Mahraz Fez Morocco.

### 2.3. Animals

To perform this study, swiss albino mice weighing 20 to 25 g were obtained from the animal center of the Faculty of Sciences Dhar El Mahraz Fez, Morocco. They were kept under monitored conditions of temperature (22 ± 2 °C), humidity (45–50%), light (12:12 h light-dark cycle), had free access to water and rodent chows. They were acclimatized for one week before starting the experiment. The care and handling of animals were carried out according to the internationally accepted standards for the use of animals [10]. Moreover, the Animal Ethics Review Committee of the Faculty of Sciences, Fez, Morocco, approved the protocol (04/2019/LBEAS).

### 2.4. Preparation of Flavonoids Extracts

After drying at room temperature in the shade, leaves of oleaster were ground into powder. Flavonoids extraction was carried out as follows; 10 g of plant powder was extracted with 100 mL of 70% methanol using an ultrasound-assisted extraction apparatus (frequency of 35 KHz) for 40 min. The extract obtained was filtered and concentrated using a rotary evaporator. The dry extract was dissolved in distilled water, then washed twice with chloroform and dichloromethane (*v*:*v*) to eliminate lipids and pigments and most of the mannitol. Afterward, the extract obtained was washed again twice with N-butanol to get the flavonoids fraction. The N-butanol phase was concentrated and stored at 4 °C until future use.

### 2.5. Evaluation of Antidiabetic Activity

#### 2.5.1. Induction of Experimental Diabetes

Alloxan monohydrate was used to provoke experimental diabetes, and to induce severe diabetes, a pretested dose of alloxan monohydrate (180 mg/kg b.w.) was intraperitoneally injected to 12 h fasted animals. In order to prevent a hypoglycemic shock after injection, a solution of 0.2 mL glucose solution (4 g/L) was orally administered. After four days, the first blood glucose measurements were taken. The animals with blood glucose levels above 450 mg/dL were selected for further study.

#### 2.5.2. The Experimental Model of Fasting Glucose Measurement

Selected mice were divided into five groups including five mice in each:

Group A: Normal mice treated with 0.2 mL/day of distilled water.

Group B: Mice with induced diabetes treated with 0.2 mL/day of distilled water.

Group C: Mice with induced diabetes treated with 2 mg/kg/day b.w. of Glibenclamide.

Group D: Mice with induced diabetes treated with 25 mg/kg/day b.w. of oleaster flavonoids.

Group E: Mice with induced diabetes treated with 50 mg/kg/day b.w. of oleaster flavonoids.

Alloxan monohydrate induces diabetes via a mechanism that basically involves partial degradation of the beta (β) cells and as a result it adversely affects the quality and quantity of insulin produced. Glibenclamide treatment was used as positive control to decrease the blood glucose concentration and increase the insulin level in both models severe and mild diabetes mellitus targeting the surviving (β) cells.

The treatments were performed using intra-gastric gavage for 28 days. Fasting blood glucose level was measured after 12h fast, within the 1st, 7th, 14th, 21st, and 28th days of treatment. Blood was collected from the tail vein, and the FBG was estimated using a glucometer (Accu-chek), whose principle is based on the glucose oxidase method.

Weight measurements were taken weekly, and at the end of the experimental period, the animals were deprived of food overnight and then sacrificed by cervical decapitation after anesthesia. Blood was collected in heparin tubes for measuring biochemical markers of liver and kidney function.

#### 2.5.3. Evaluation of the Antihyperglycemic Activity

After the first measure on the 1st day and selection of groups, the treatments were administered right away. A series of blood glucose level measurements were conducted after 1 h, 1 h 30 min, 3 h, 6 h, and 12 h of treatment administration for the groups which have taken the Oleaster flavonoids and Glibenclamide to see how those treatments will manage the hyperglycemic status of the mice during this period.

### 2.6. Oleaster Flavonoids Qualitative Analysis

Qualitative profiling of oleaster leaves flavonoids was made by ultra-high-performance liquid chromatography with triple quadrupole mass spectrometry. The extracts were resuspended in 2 mL of a solution of water:acetonitrile 1:1. Twenty microliters of these solutions were diluted in 980 microliters of acetonitrile and injected for LC-MS/MS analysis. An in-house developed database comprising the secondary metabolites of polyphenols was used for the purpose of screening, and each phenolic compound was identified based on the detection of the precursor ion, and at least one characteristic fragment ion.

The analysis was performed using a Shimadzu Ultra-High-Performance Liquid Chromatography (UHPLC; Nexera XR LC 40), coupled to an MS/MS detector (LCMS 8060, Shimadzu Italy, Milan, Italy). The MS/MS was operated with electrospray ionization (ESI) and controlled by Lab Solution software, which simultaneously provided quick switching from low energy scan at 4 V (full scan MS) to high energy scan (10–60 V ramping) during a single LC run. The low-CE experiments provided information about the intact molecular ion (e.g., M^+^, [M + H]^+^), while the high-CE scan generates fragment ion information. The source parameters were set as follows: nebulizing gas flow 2.9 L/min, heating gas flow 10 L/min, interface temperature 300 °C, DL temperature 250 °C, heat block temperature 400 °C, drying gas flow 10 L/min.

Qualitative analysis was performed in flow injection with a mobile phase consisting of acetonitrile:water + 0.01% formic acid (5:95, *v*/*v*).

### 2.7. Molecular Docking

#### 2.7.1. Preparation of Ligands

Based on the LC/MS results, five ligands were selected for docking simulation. The SDF file of each ligand was retrieved from PubChem (Amentoflavone, CID: 5281600; Quercetin, CID: 5280343; Rutin, CID: 5280805; luteolin-7-O-glucoside, CID: 45933934; Oleuropein, CID: 5281544.) Such files could not be directly used to dock simulation, thus utilizing AutoDockTools v1.5.6 [11], and therefore they were converted to PDBQT format. Non-polar hydrogen atoms were merged, Gasteiger partial charges were added, and rotatable bonds were defined.

#### 2.7.2. Preparation of Receptors

The X-ray crystal structures of the eight receptors were retrieved from the Protein Data Bank (PDB)) [12] (PTP1B, PID: 1c83; DPP4, PID:2p8s; FFAR1, PID:4phu; Alpha amylase, PID: 1smd; PPAR gamma, PID:5ycp; Alpha glucosidase, PID: 5nn5; Aldose reductase, PID:2hv5; Glycogen phosphorylase, PID:1l5q). The structure was edited to remove water, ligands, and heteroatoms (HETATM) using Discovery Studio Visualizer v 19.1.0 (BIOVIA, San Diego, CA, USA). Preparation of receptor files involves changing atom type, removing water molecules, adding polar hydrogen atoms, Gasteiger charges, and conversion into PDBQT format using AutoDockTools v1.5.6.

#### 2.7.3. Docking Simulations

Molecular docking studies were carried out for the selected molecules in the binding site of target proteins using AutoDock Vina [13] and AutoDock Tools.

The grid box size was set for each receptor, and the exhaustiveness was set to 24. The results with the best conformation and energy were selected for further analysis. Discovery Studio Visualizer V19 was used for visualization and analysis of the protein-ligand complexes.

## 3. Results and Discussion

### 3.1. Yield of Extraction

The extraction yield of flavonoids from leaves of oleaster was 5.7%.

### 3.2. Oleaster Flavonoids Qualitative Analysis

**Figure 1:**
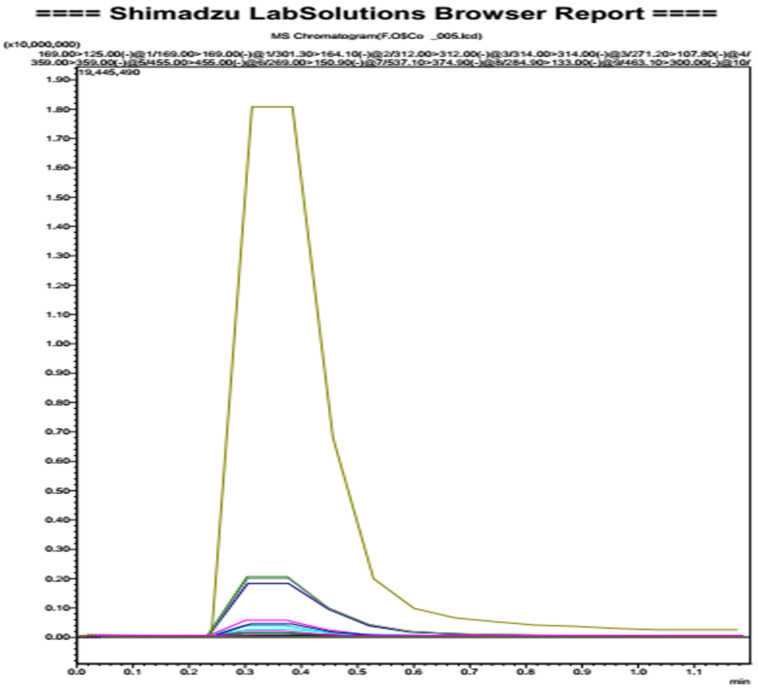
LC/MS-MS qualitative results of oleaster flavonoids.

The identification was carried out according to the molecular weight of the typical fragments of molecules. The analysis revealed the presence of five molecules belonging to the flavonoid family (Amentoflavone, Quercetin-3-*O*-glucoside, Quercetin-3-*O*-hexose-deoxyhexose, luteolin-7-*O*-glucoside, Rutin). The 6th molecule was Oleuropein which belongs to secoiridoid as a characteristic molecule of the Olearaceae family (Figure 1 and Table 1).

### 3.3. Antidiabetic Activity

#### 3.3.1. Effect of Oleaster Flavonoids Extract on Glycemia in Diabetic Mice

**Figure 2:**
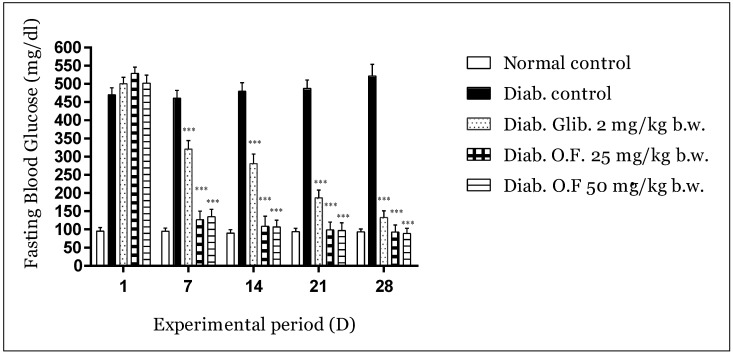
Effect of flavonoids on fasting blood glucose Alloxan-induced diabetic mice during the experimental period of 28 days. Values are expressed as mean ± SD (*n* = 5 mice). *** *p* < 0.001 compared to diabetic control.

Figure 2 displays the impact of the treatments on fasting blood glucose levels (weekly measurement).

Within the first week, reduction reached 76% due to oleaster leaves flavonoids at a dose of 25 and 73% for a dose of 50 vs. 36% for Glibenclamide compared to the negative control group (*p* < 0.001). Hyperglycemia was suppressed afterward, and the FBG was significantly maintained (*p* < 0.001) with a reduction of 79, 79 and 44% (second week), 81, 81 and 63% (third week), 82, 82 and 73% (fourth week) respectively, compared to the negative diabetic control. The antidiabetic activity of the extract was better even than that obtained with Glibenclamide and reached normal glycemic values in the last week (normal mice glycemia: 93.7 mg/dL; mice treated with O.F. 25 mg/kg: 93 mg/dL; mice treated with 50 mg/kg: 89 mg/dL).

The bodyweight development and biochemical parameter of the different experimental mice groups are represented in Table 2. Consecutive decline in body weight was observed in the untreated diabetic group compared to normal animals. On the other hand, the groups treated with oleaster flavonoids for four weeks showed a very significant increase compared to the diabetic control group (*p* < 0.001). Hyperglycemia leads to body weight loss due to the intense usage of fats and structural proteins as a replacement for carbohydrates [14], which its usage and regulation of depend on insulin [15]. Oral administration of oleaster flavonoids on the first degree and Glibenclamide at a second degree for four weeks to diabetic mice improved their body weight and managed their hyperglycemic state.

The results obtained showed that significant increase in the activities of ALAT and ASAT (liver biomarkers) was observed in the untreated diabetic animals compared to normal animals (*p* < 0.001). However, the plasma level of transaminases was remarkably reduced in the blood of diabetic animals after treatment with oleaster flavonoids (*p* < 0.001). Our study was in agreement with earlier data which showed that oleaster flavonoids exhibiting a remarkable hepatoprotective effect against possible damage that may be caused by the injection of Alloxan monohydrate [16]. Alloxan-induced diabetic mice also induced a significant increase in urea and creatinine after four weeks of treatment compared to normal animals (*p* < 0.001). However, the treatment of diabetic mice with oleaster flavonoids significantly decreased their urea and creatinine levels in comparison with the untreated diabetic animals (*p* < 0.01). Elevated levels of urea and creatinine are considered markers for renal damages [17]. Hence, elevated urea and creatinine levels observed in diabetic mice indicate impaired renal function. In comparison, the administration of oleaster flavonoids to the diabetic mice greatly decreased creatinine and urea levels, and they are suggested to prevent kidney damages in diabetic conditions.

#### 3.3.2. Antihyperglycemic Activity of Oleaster Flavonoids on Diabetic Mice

**Figure 3:**
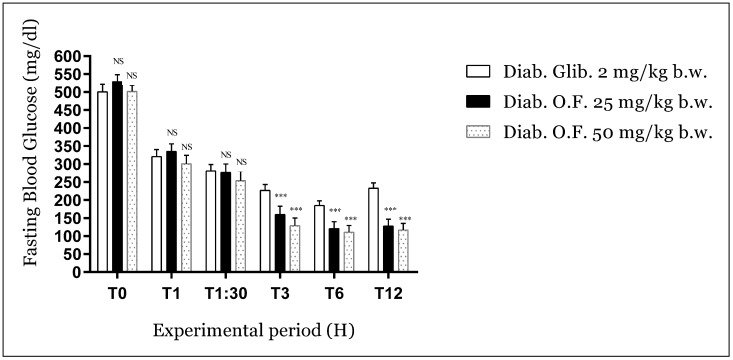
Effect of oleaster leaves on hyperglycemic status of Alloxan-induced diabetic mice during the experimental period of 12 h. Values are expressed as mean ± SD (*n* = 5 mice). NS: non-significant; *** *p* < 0.001 compared to diabetic control.

The antihyperglycemic activity of oleaster flavonoids is represented in Figure 3. During the experimental period of 12 h, the obtained results showed that no significant reduction in blood glucose value of treated mice within the first hour and an hour and a half compared to the positive control (Glibenclamide). The reduction started beginning after 3 h (70, 75, and 55%, for oleaster flavonoids 25.50 and Glibenclamide respectively) and at the 6th hour (77, 78 and 63%, for oleaster flavonoids 25.50 and Glibenclamide respectively) (*p* < 0.001). After 12 h of treatment, glucose values in plasma of positive control mice significantly increased compared to groups treated with oleaster flavonoids (76, 77, and 55%, for oleaster leaves flavonoids 25.50 and Glibenclamide respectively) (*p* < 0.001). These results may reflect a long-term effect and the powerful management of the hyperglycemic status of diabetic mice.

## 4. Molecular Docking

The selected receptors for this study are highly involved in diabetes manifestations or management. Only the binding poses of ligands with the highest affinities and bonds with known active sites were selected and analyzed. Affinity results are summarized in Table 3.

It’s important to note that in the molecular docking simulations, we replaced both quercetin-3-*O*-glucoside and quercetin-3-*O*-hexose-deoxyhexose with quercetin as the attached sugar moieties were removed after ingestion before absorption can take place [18].

### 4.1. PTP1B

Protein tyrosine phosphatase 1B (PTP1B) is an important enzyme in insulin receptor dephosphorylation [19]. Genetic deletion of PTP1B in mice further established the essential functions of PTP1B linked to diabetes and obesity. PTP1B-deficient mice remained insulin sensitive and were resistant to weight gain on a high-fat diet [19,20]. For PTP1B, the catalytic domain includes the active site Cys215 and catalytic loop consisting of His214, Ser216, Ala217, Gly218, Ile219, Gly220, and Arg221 [21,22], and Trp179, Pro180, and Asp181 [23].

According to the docking results, Luteolin-7-*O*-glucoside has the highest affinity to PTP1B. This finding indicates that oleaster flavonoids exhibit their antidiabetic activity via inhibition of PTP1B. Previous research indicates that the eradication or reduction of PTP1B improves glucose tolerance and the regulation of insulin. PTP1B inhibition also decreased the concentration of triglycerides in the adipose tissue under over-nutrition conditions and was not associated with any apparent toxicity [24].

### 4.2. DPP4

Dipeptidyl-peptidase 4 (DPP4) is a glycoprotein found on several cell surfaces. It selectively cleaves N-terminal dipeptides from various substrates like growth factors, cytokines neuropeptides, and the incretin hormones (glucagon-like peptide-1 and glucose-dependent insulinotropic polypeptide (major regulators of postprandial insulin secretion) [25].

The active site of DPP4 in its entirety is considered to contain residues 39–51 and 501–766 and is known as the α/β-hydrolase domain [26,27]. The docking affinities of the studied compounds to the DPP4 active site are quite high with amentoflavone showing the strongest binding. High docking affinities indicate that DPP4 inhibition is one of the major actions of all oleaster flavonoids. DPP4 inhibition is characteristic of the gliptin family of drugs that have gained significant popularity in Type 2 diabetes therapy.

### 4.3. FFAR1

Human G-protein coupled receptor 40 (hGPR40), also known as free fatty acid receptor 1 (FFAR1), is a seven helical transmembrane domain receptor that recognizes long-chain free fatty acids and induces insulin secretion [28]. FFAR1 is highly expressed in human pancreatic β cells, brain, and endocrine cells of the gastrointestinal tract [29].

Free fatty acids bind to FFAR1 by coordinating their free carboxyl group to three amino acids, Arg183, Tyr2240, and Arg258, which are located close to the extracellular domain [30]. hGPR40 has a distinct binding pocket that is established by eight key residues: Tyr91, Glu172, Arg183, Ser187, Tyr240, Asn241, Asn244, and Arg258 [31,32]. Docking analysis has noted that only two ligands (Luteolin-7-*O*-glucoside and Oleuropein) have affinities for the active site. Even though only two ligands have an affinity to the FFAR1 active site, activation of this protein remains one of the possible modes of action of the oleaster flavonoids. FFAR1 is of particular interest because the triggering of insulin secretion is glucose-dependent, which makes it a major focus for the treatment of type 2 diabetes, as agonists will improve glycemic regulation and minimize the risk of hypoglycemia [28].

### 4.4. α-Amylase

α-Amylase (AAM) is an enzyme that works as a catalyst for the hydrolysis of α-linked polysaccharides into α-anomeric products [33]. α-Amylase is actively found in pancreatic juice and saliva [34].

The AAM active site contains three catalytic residues: Asp197, Glu233, and Asp300. Many residues are also important for the enzyme activity: Arg337, Arg195, Asn298, Phe265, Phe295, His201, Ala307, Gly306, Trp203, Trp284, Trp59, Tyr62, Trp58, His299, His101 [35,36,37,38].

All five tested ligands have an affinity to the active site. The highest affinity was noted for Amentoflavone, and Oleuropein has the lowest among the studied series. According to our findings, α-amylase inhibition is also one of the major modes of action of the oleaster flavonoids as all the ligand exhibit activity toward it. α-amylase is a part of the digestion associated with carbohydrate breakdown.

### 4.5. PPARγ

Peroxisome proliferator-activated receptor gamma (PPAR-γ or PPARG), also known as the glitazone receptor, plays his role by enhancing the storage of fatty acids in fat cells, which increase insulin sensitivity and reduce lipotoxicity [39].

The PPARγ ligand-binding domain is folded into a helical sandwich to provide a binding site for ligands. It is located at the C-terminal end of PPARγ. It is composed of about 250 amino acids, activation by full agonists occurs through hydrogen bond interactions between the Ser289, His323, Tyr473, and His449 residues of the PPARγ ligand-binding domain [40].

Detailed analysis of the active site of PPARγ reveals that none of the ligands could bind with it. Reference molecule Rosiglitazone (an antidiabetic drug of the thiazolidinedione class) has shown that this molecule binds perfectly with the active site. These findings indicate that PPARγ is not involved in the global mode of action of oleaster flavonoids.

### 4.6. α-Glucosidase

Alpha-glucosidase enzymes catalyze the hydrolysis of starch and dietary carbohydrates to simple sugars (glucose) for intestinal absorption, which, in turn, results in increased blood glucose levels [41].

The residues involved into the activity of the enzyme are Asp404, Asp518, Arg600, Asp616, and His674 Trp376, Ile441, Tp516, Met519, Trp613, and Phe649 Leu405, Trp481, Asp645, and Arg672 [42,43].

Good affinities with the active site were noted for all ligands. The highest affinity was observed for Amentoflavone. It corroborates that α-glucosidase inhibition can be one of the major modes of action of the oleaster flavonoids. Inhibiting the function of this enzyme may help reduce postprandial hyperglycemia.

### 4.7. Aldose Reductase

Aldose reductase (AldR), a key enzyme in the polyol pathway or sorbitol-aldose reductase pathway, catalyzes NAD(P)H-dependent reduction of glucose to sorbitol, leading to excessive accumulation of intracellular reactive oxygen species (ROS) in various tissues [44], that may lead to very serious health problems [45]. Aldose reductase structure is of a β/α-barrel. The active site is located in the barrel core [46]. All five ligands have demonstrated an excellent affinity for the active site. The highest affinity characterized amentoflavone. Aldose reductase inhibition is expected to be one of the keys preventing hyperglycemia by the oleaster flavonoids extract as all five studied ligands manifested its inhibition.

### 4.8. Glycogen Phosphorylase

Glycogen phosphorylase (GP) catalyzes the hydrolysis of glycogen to generate glucose-1-phosphate [47]. The 280’s loop (residues 280–288), which lies between β-strand 11 and α-helix 8, is thought, in particular, to play a key role in the control of substrate and inhibitor binding [48,49].

Only four ligands have shown affinities for the active site. The highest affinity was again noted for Amentoflavone. Glycogen phosphorylase is considered as one of the causes of hyperglycemia as it is responsible for generating glucose. Inhibiting this enzyme by different ligands present in oleaster flavonoids may lead to considerable improvement of the glycemic status of diabetic people.

Among all the ligand tested, it is obvious that Amentoflavone is the most polyvalent molecule with the best affinities to almost all selected receptors, followed by Luteolin-7-*O*-glucoside, Rutin, and Quercetin. Oleuropein had the lowest binding affinities among the studied series.

## 5. Conclusions

Besides its nutritional and cosmetic values, the wild olive tree (oleaster) presents a promising source of bioactive molecules that can be exploited to fight some diseases. This work provides big data on the antidiabetic activity, the safety as well as the potential mode of action of oleaster flavonoids using molecular docking. *Olea europea* exhibited very interesting results in diabetic control as well presented in this work, and therefore we could confirm that oleaster flavonoids could be a game changer in the treatment of diabetes.

## Figures and Tables

**Table 1:** Oleaster flavonoids displayed by LC/MS-MS.

Molecule	Analyzed Fragment	Area under Curve
Amentoflavone	537.10 > 374.90	88,078
Quercetin-3-*O*-glucoside	463.10 > 300.00	840,585
Quercetin-3-*O*-hexose-deoxyhexose	609.10 > 300.00	1,426,485
luteolin-7-*O*-glucoside	447.10 > 285.00	21,618,792
Oleuropein	539.00 > 539.00	195,134,071
Rutin	609.00 > 301.00	1,000,877

**Table 2:** Effect of flavonoids from oleaster leaves on bodyweight development and biochemical parameters of Alloxan-induced diabetic mice during the experimental period of 28 days.

Treatments	Bodyweight Development					Biochemical Parameters			
	Day 1	Day 7	Day 14	Day 21	Day 28	ASAT (UI/L)	ALAT (UI/L)	UREA (g/L)	CREATININE (mg/L)
**Normal Control** **DW 2 mL/day**	23.4 ± 1.8 ^ns^	24.7 ± 1.5 *	25.2 ± 1.4 ***	26.9 ± 1.3 ***	27.2 ± 1.5 ***	253 ± 23.7 ***	45 ± 7.5 ***	0.24 ± 0.04 ***	3.2 ± 0.4 ***
**Diab. Control** **DW 2 mL/day**	23.8 ± 1.6	21.7 ± 2.2	20.1 ± 2.7	19.2 ± 2.5	17.7 ± 2.4	502 ± 38.8	134 ± 11.2	0.63 ± 0.06	5.8 ± 0.8
**Diab. Glib.** **2 mg/kg/day**	24.3 ± 1.6 ^ns^	23.1 ± 1.5 ^ns^	24.2 ± 1.7 **	25.8 ± 1.8 ***	25.9 ± 1.7 ***	298 ± 24.6 ***	77 ± 8.8 ***	0.28 ± 0.04 ***	4.2 ± 0.4 **
**Diab. O.F.** **25 mg/kg/day**	23.7 ± 1.2 ^ns^	24.1 ± 1.3 ^ns^	25.8 ± 1.1 ***	26.6 ± 1.2 ***	28.4 ± 1.1 ***	223 ± 20.1 ***	51 ± 7.0 ***	0.35 ± 0.03 ***	3.6 ± 0.5 **
**Diab. OF.** **50 mg/kg/day**	23.5 ± 1.9 ^ns^	24.5 ± 2.3 ^ns^	26.1 ± 1.9 ***	27.6 ± 2.2 ***	28.9 ± 2.4 ***	286 ± 18.7 ***	44 ± 5.5 ***	0.30 ± 0.03 ***	3.8 ± 0.4 **

Values are expressed as mean ± SD (*n* = 5 mice). ns: non-significant; * *p* < 0.05 ** *p* < 0.01 *** *p* < 0.001 compared to diabetic control.

**Table 3:** Summary of affinities of different ligands with the receptors.

	Affinity (kcal/mol)							
	PTP1B	DPP4	FFAR1	Alpha Amylase	PPAR Gamma	Alpha Glucosidase	Aldose Reductase	Glycogen Phosphorylase
Amentoflavone	−8.8	−10.5	None	−11.3	None	−9.5	−10.0	−10.7
Quercetin	−6.9	−8.7	None	−8.4	None	−7.3	−9.6	−9.8
Rutin	−7.3	−9.2	None	−8.4	None	−8.1	−8.3	−8.4
Luteolin-7-*O*-glucoside	−8.9	−9.1	−8.2	−10.0	None	−8.5	−8.8	−8.2
Oleuropein	−6.6	−7.3	−6.2	−6.9	None	−7.2	−9.1	None

2D view of ligand-receptor interactions are presented in the Supplementary Materials.

## Supplementary Materials

The following are available online: 2D view of ligand-receptor interactions.

## Abbreviations

BGL	Blood glucose level
GLIB	Glibenclamide
DW	distilled water
PTP1B	Protein tyrosine phosphatase 1B
DPP4	Dipeptidyl-peptidase 4
AAM	α-Amylase
AldR	Aldose reductase
GP	Glycogen phosphorylase
FFAR1	Free fatty acid receptor 1
ROS	Reactive oxygen species
