# Supplementary material for: In-Vivo Antidiabetic Activity and In-Silico Mode of Action of LC/MS-MS Identified Flavonoids in Oleaster Leaves
Source: Molecules. 2020 Nov 1;25(21):5073. doi: 10.3390/molecules25215073 (PMC7663640; doi:10.3390/molecules25215073)
Supplement: Supplementary file 1 [file molecules-25-05073-s001.pdf]

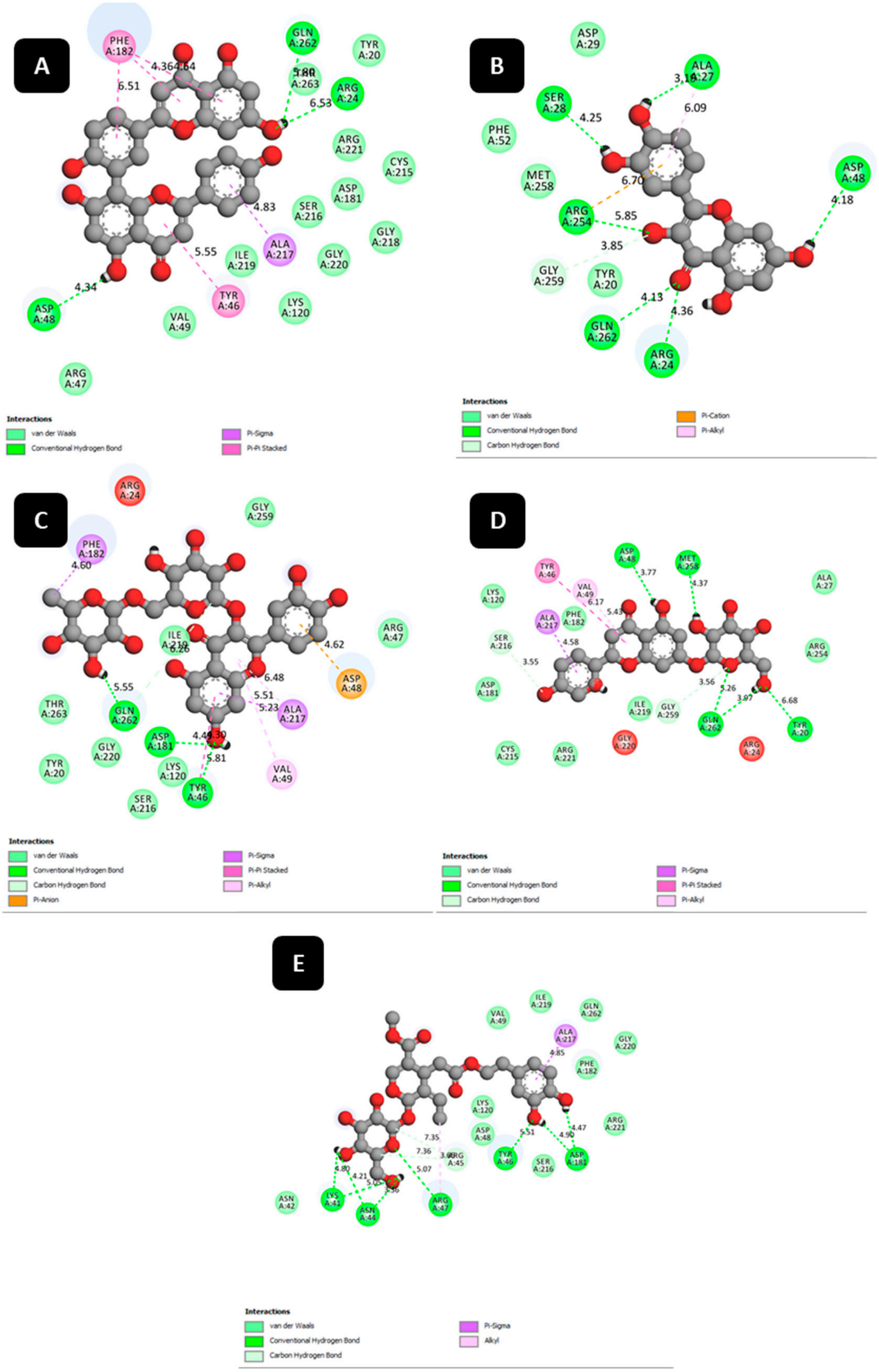


**2D view of the ligand interactions with PTP1B receptor (A: Amentoflavone; B: Quercetin; C: Rutin; D: luteolin-7-O-glucoside; E: Oleuropein).**

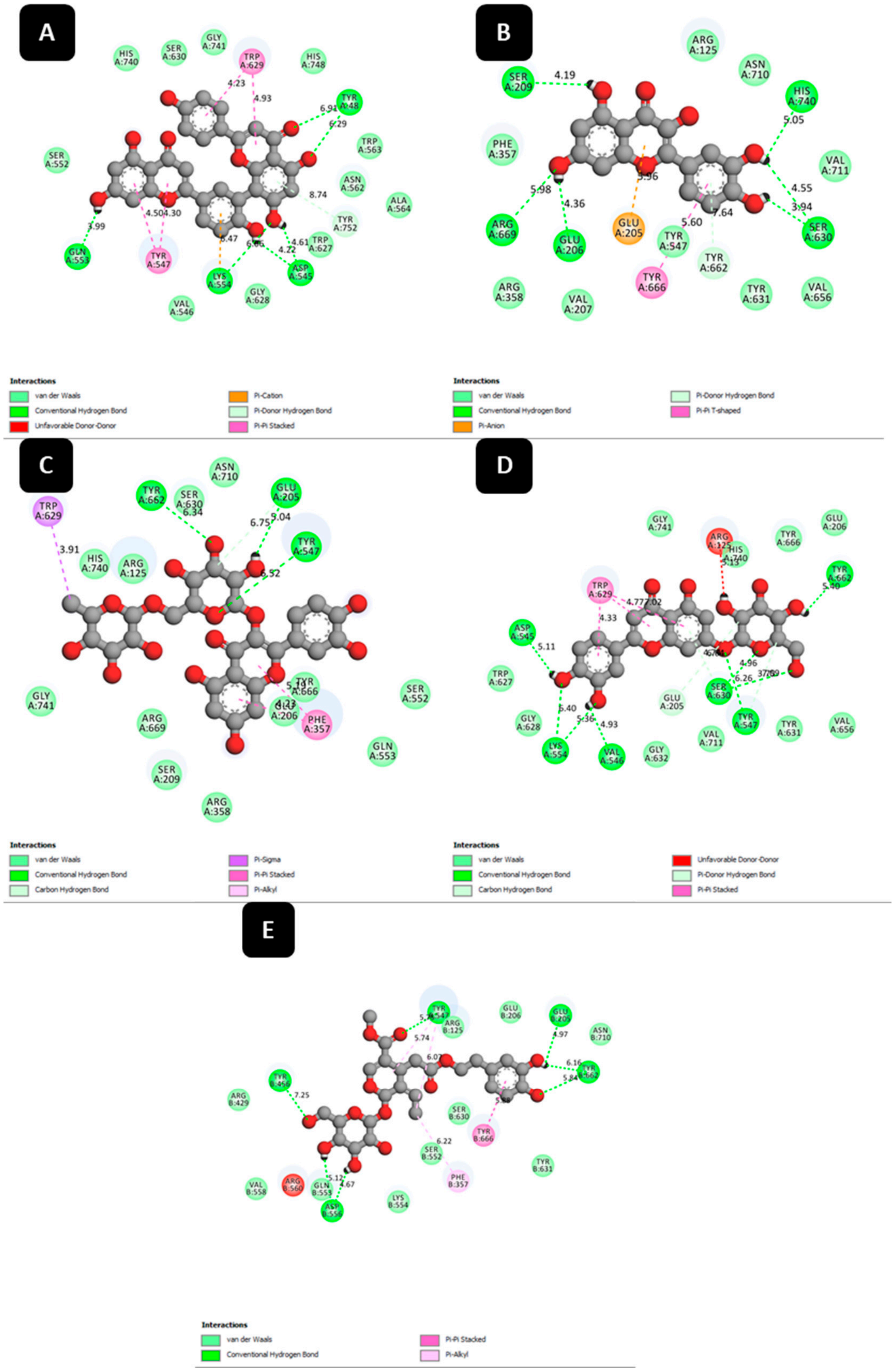


**2D view of the ligand interactions with DPP4 receptor (A: Amentoflavone; B: Quercetin; C: Rutin; D: luteolin-7-O-glucoside; E: Oleuropein).**

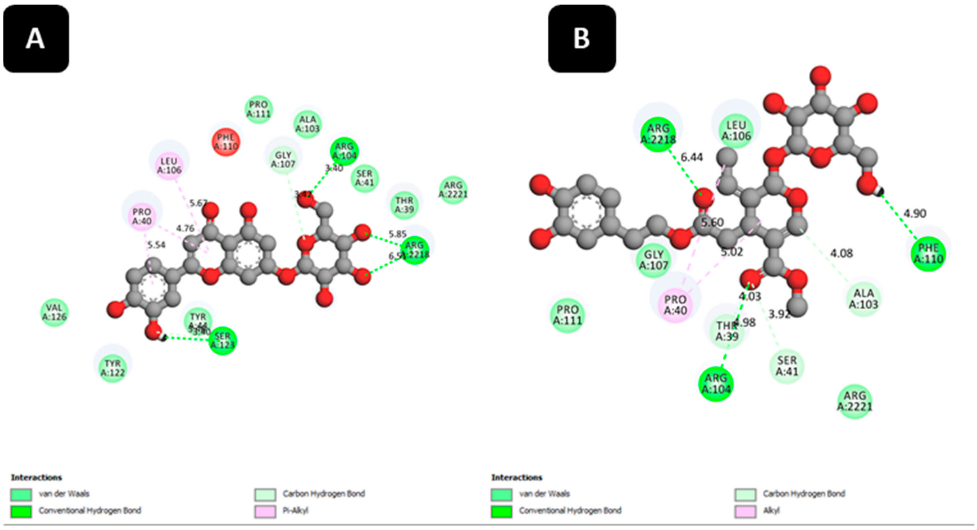


**2D view of the ligand interactions with FFAR1 receptor (A: luteolin-7-O-glucoside; B: Oleuropein).**

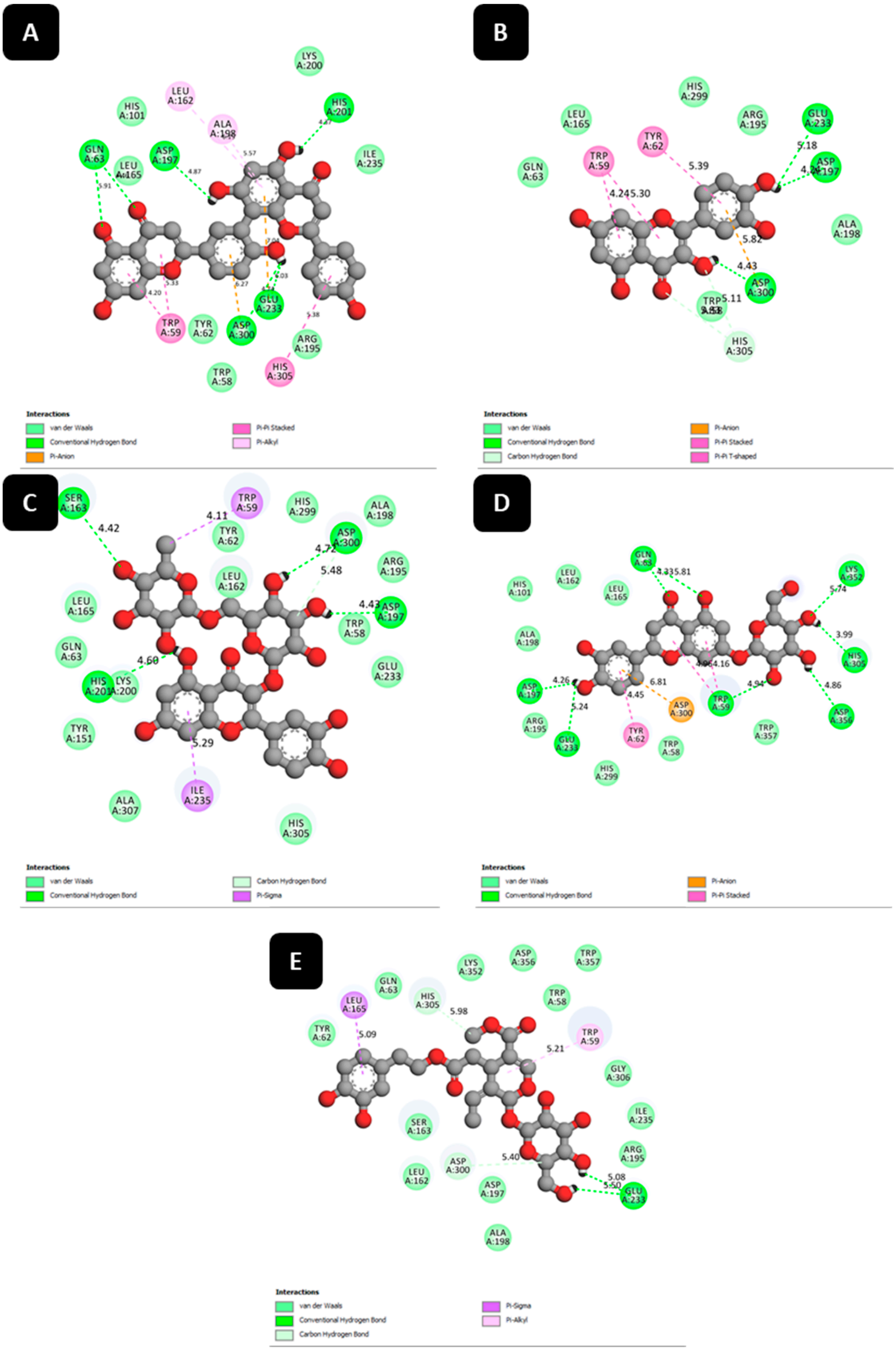


**2D view of the ligand interactions with Alpha amylase receptor (A: Amentoflavone; B: Quercetin; C: Rutin; D: luteolin-7-O-glucoside; E: Oleuropein).**

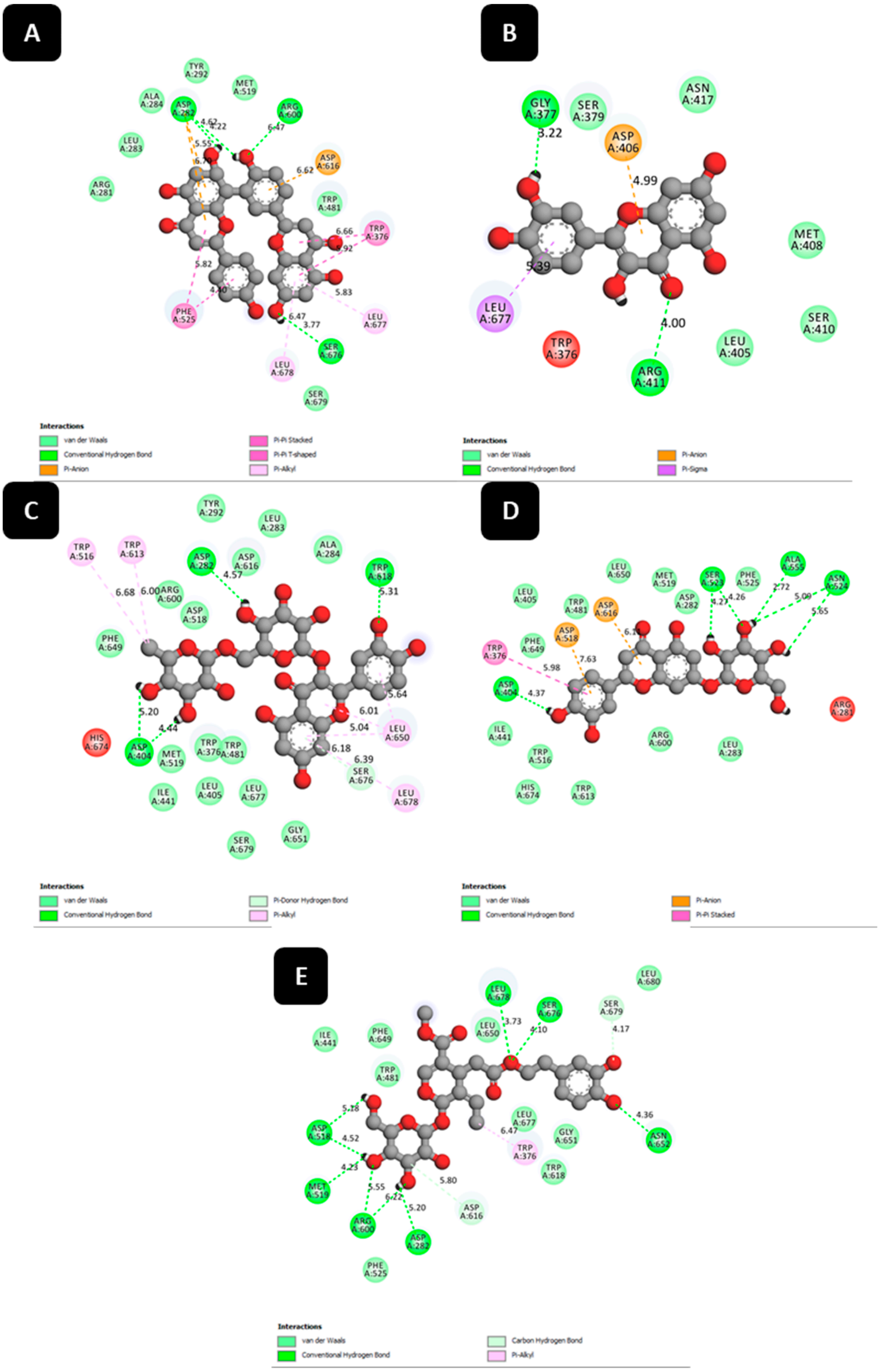


**2D view of the ligand interactions with alpha glucosidase receptor (A: Amentoflavone; B: Quercetin; C: Rutin; D: luteolin-7-O-glucoside; E: Oleuropein).**

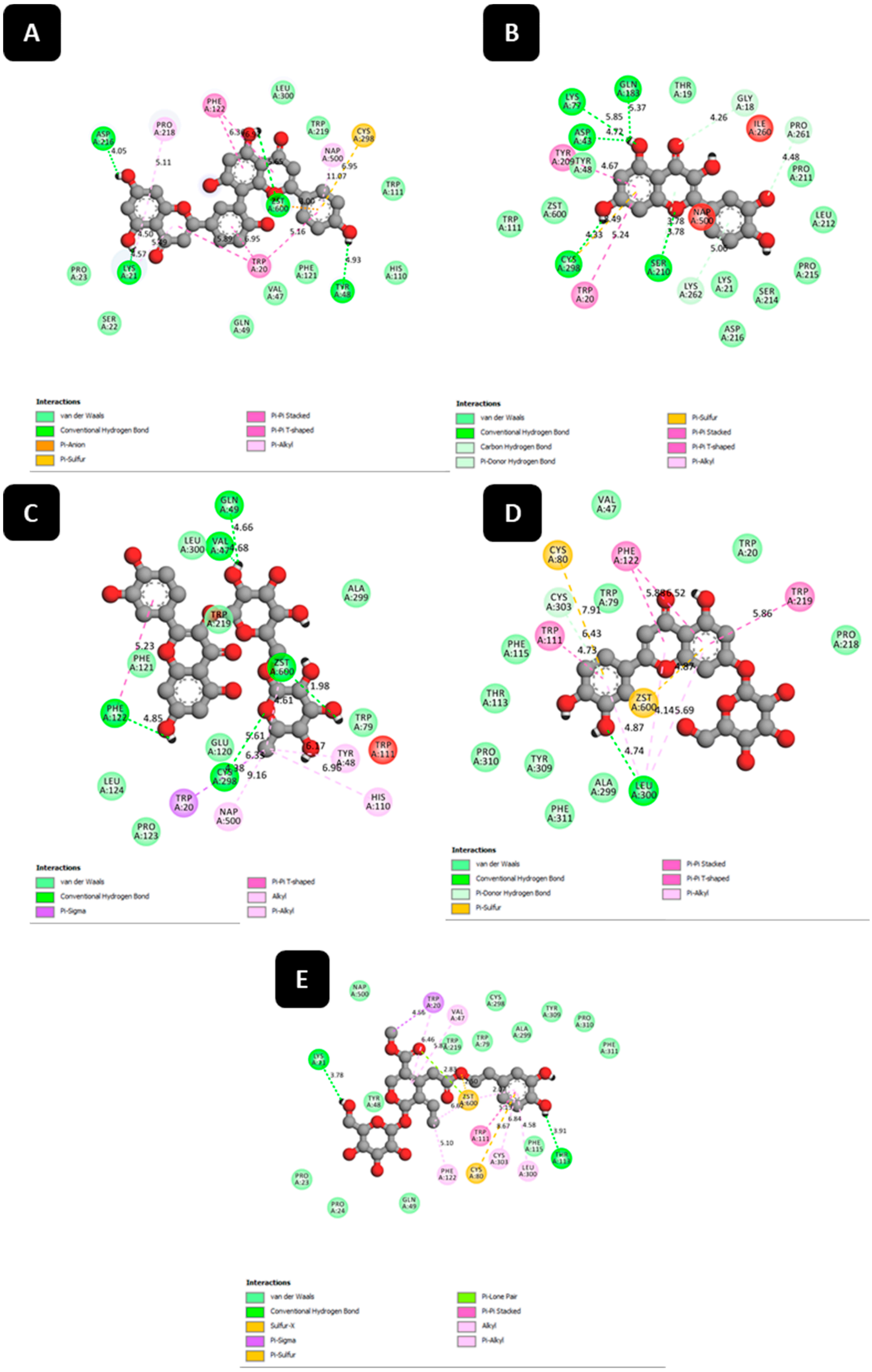


2D view of the ligand interactions with Aldose reductase receptor (A: Amentoflavone; B: Quercetin; C: Rutin; D: luteolin-7-O-glucoside; E: Oleuropein).

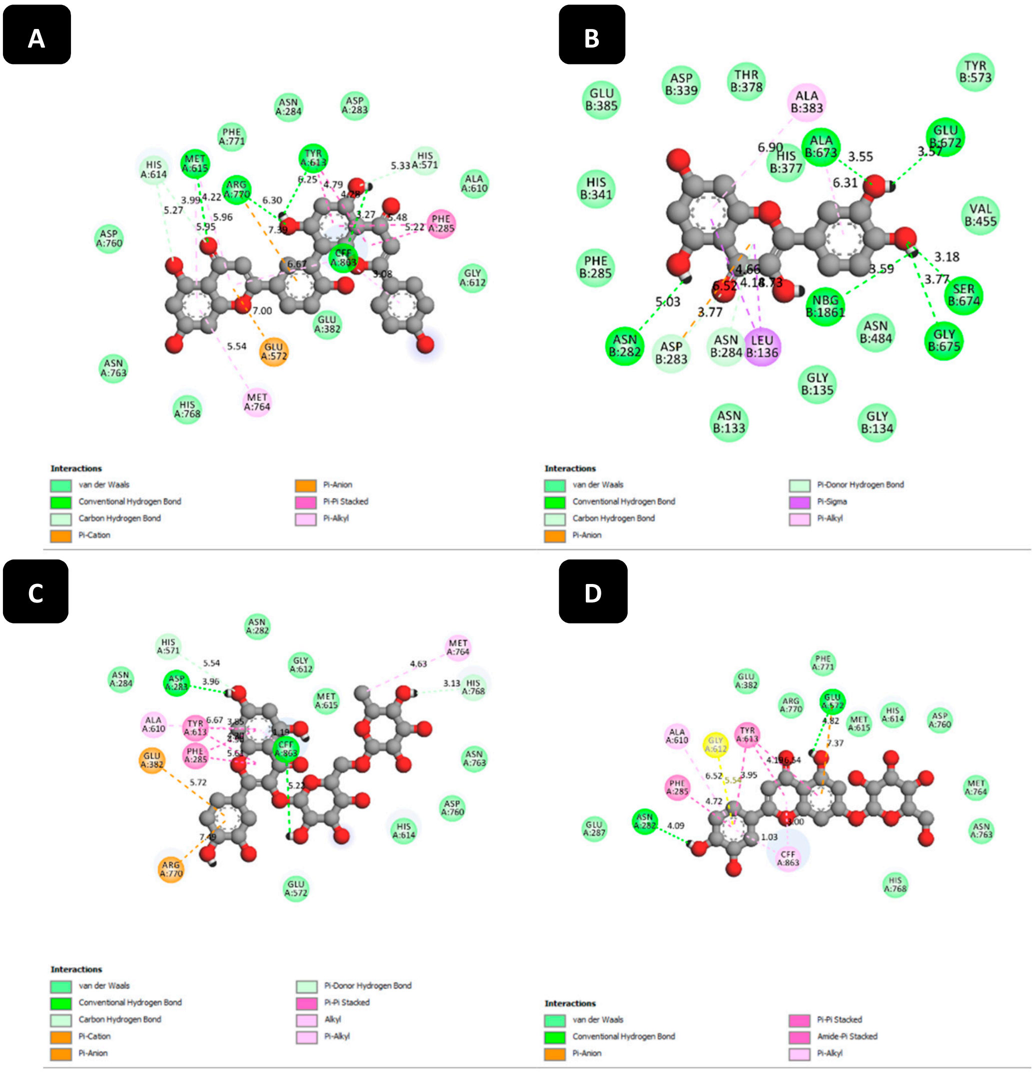


**2D view of the ligand interactions with Glycogen phosphorylase receptor (A: Amentoflavone; B: Quercetin; C: Rutin; D: luteolin-7-O-glucoside;).**
